# Making cytology specimens solid materials for testing predictive marker of immunotherapy in NSCLC

**DOI:** 10.18632/oncotarget.26261

**Published:** 2018-10-26

**Authors:** Hangjun Wang, Alan Spatz

**Affiliations:** Division of Pathology, Jewish General Hospital and McGill University Health Center, Montreal, Quebec, Canada

**Keywords:** cytology cell block, PD-L1 testing

Immunotherapy has significantly changed the standard care in non-small cell lung cancer (NSCLC). Immune check point inhibitors including Pembrolizumab, Nivolumab, Atezolizumab or Durvalumab have been approved for either first line, second line treatment or combined with other treatment. In Canada, for first-line treatment by Pembrolizumab, high PD-L1 expression (Tumor Proportion Score TPS ≥ 50%) is required for patients with metastatic NSCLC without targetable gene alteration. Accordingly, it becomes critical to determine PD-L1 expression in addition to pathological diagnosis of NSCLC. Diagnostic kits such as PD-L1 IHC 22C3PharmDx assay or PD-L1 IHC 28-8 PharmDx assay have been approved by FDA and used in clinical trials. These assays were evaluated on biopsy or resection specimens only, but not on cytology materials.

The reason for not including cytology specimens in the clinical trials was probably the customary immunohistochemistry staining on tissue sections, and unfamiliarity and concern of inadequacy of cytology specimens. However, comparing to biopsy, cytology sampling is less invasive. NSCLC cytology materials can be obtained by fine needle aspirations (FNA), bronchoalveolar lavages (BAL) or aspiration of pleural/pericardial effusion. Some of the procedures such as endobronchial ultrasound-guided (EBUS) FNA have been used for both diagnosis and staging. EBUS has often replaced traditional mediastinal lymph node biopsy by mediastinoscopy. Cytology procedures can be combined with rapid on-site evaluation (ROSE) for the evaluation of adequacy. The specimens can be prepared as direct smears, liquid-based cytology (Cytospin or ThinPrep or SurePrep) or cell blocks. Cell blocks are processed as formalin-fixed paraffin-embedded (FFPE) similar to histological tissue specimens. For *ALK* immunohistochemical staining, cytology cell block has been well validated and deemed as appropriate materials in 2018 CAP/IASLC/AMP guidelines [1].

Therefore, many patients were either deprived from immunotherapy or had to undergo invasive biopsy. Recently, studies assessing cytology materials for PD-L1 immunocytochemical have started to emerge [2-6]. For PD-L1 testing, a high concordance was found between paired cytologic cell block and histologic material from the same site of NSCLC.

Taking the advantage of available materials as a reference testing center, we have recently performed a large study to assess the feasibility using cytology cell blocks for PD-L1 immunostaining [7]. In our study, a total of 371 cytology cell blocks were evaluated in comparison with 809 small biopsies and 239 surgical resections. The cytology specimens included EBUS-guided FNA, endoscopic ultrasound-guided FNA, FNA, pleural/pericardial fluid and bronchoalveolar lavage. We observed that the fail rate of cytology cell block was 8% similar to biopsy. When using TPS ≥ 50% as cutoff for high expression, the rate of high PD-L1 expression in cytology specimens was similar to small biopsy, but significantly higher than in surgical resection specimens. We compared the fixation methods and did not find any difference of PD-L1 expression between the formalin, combined Cytolyt and formalin, and Cytolyt/alcohol only groups. We found that advanced stages were associated with higher PD-L1 TPS. Similar to the findings from Torous et al [6], additional clinical follow-up of small portion of our patients treated with immunotherapy reveal that there was no significant difference in response and disease control rates between cytology and small biopsy groups. These findings supports that PD-L1 testing on cytology cell blocks and on small biopsies similar clinical significance (data was presented IASLC 2018 World Conference on Lung Cancer Toronto).

The main disadvantage of cytologic cell block is the lack of architecture and tissue fragmentation which can make the interpretation very challenging. In our experience, the careful evaluation of nuclear features and the comparison with Hematoxylin & Eosin (H/E) slides is the most useful approach. In addition, immunohistochemical stains of BerEp4 or TTF-1 can also be used to identify tumor cells. The rare persisting difficulties should be solved by consensus. The intra-observer agreement is excellent with over 88% and 96 % for TPS cutoff at 1% and 50% respectively [4]. The discrepancies were related to the heterogeneous expression of PD-L1 in tumors.

Currently, most of the experience with PD-L1 immunohistochemical staining on cytology specimens comes from cell blocks. However, cell blocks are not always available and sometimes direct smears or liquid-based cytology preparation may be the only tumor specimens. Some labs started to optimize PD-L1 immunostaining on direct smears by using a procedure derived from the commercial kit [8].

More recently, tumor mutational burden (TMB) has emerged as a predictive marker of response to immune check point inhibitors in NSCLC patients [9]. Progression-free survival was significantly longer with first-line treatment by Nivolumab plus ipilimumab than with chemotherapy among patients with NSCLC and high TMB (>10 mutations per megabase). Currently, TMB testing on tumor tissue is based on next generation sequencing (NGS) technology performed on formalin-fixed paraffin-embedded (FFPE) tissue blocks. Cytology cell blocks are FFPE specimen and similar to histological tissue blocks; their use for NGS has been validated. Other types of preparations like direct smear, cytospin and liquid-based cytologies were also found feasible and suitable for NGS platform [10]. In general, as long as the cytology samples contain adequate tumor cells, it seems there is no differences between the types of procedures or cytology preparation.

In conclusion, cytologic cell blocks are evaluated as valuable and suitable specimens for PD-L1 immunohistochemical staining. We expect that cytology specimens including all types of preparation will be increasingly used for TMB testing. However, more studies are needed to further address and standardize preanalytic and analytic factors such as type and duration of fixation, different specimen preparation and the concordance between pathologists.
